# Microglia-derived TNF-α contributes to RVLM neuronal mitochondrial dysfunction via blocking the AMPK–Sirt3 pathway in stress-induced hypertension

**DOI:** 10.1186/s12974-023-02818-6

**Published:** 2023-06-01

**Authors:** Linping Wang, Tianfeng Liu, Xueping Wang, Lei Tong, Gaojun Chen, Shumin Zhou, Haili Zhang, Haisheng Liu, Wen Lu, Guohua Wang, Shuai Zhang, Dongshu Du

**Affiliations:** 1 School of Environmental and Chemical Engineering, Shanghai University, Shanghai, China; 2 College of Life Sciences, Shanghai University, Shanghai, China; 3 International Cooperation Laboratory of Molecular Medicine, Academy of Chinese Medical Sciences, Zhejiang Chinese Medical University, Hangzhou, Zhejiang China; 4 College of Agriculture and Bioengineering, Heze University, Heze, Shandong China; 5 Shaoxing Institute of Shanghai University, Shaoxing, Zhejiang China; 6 Department of Neurophysiology and Neuropharmacology, Institute of Special Environmental Medicine and Co-Innovation Center of Neuroregeneration, Nantong University, Nantong, 226019 Jiangsu China

**Keywords:** Microglia-derived TNF-α, Mitochondrial dysfunction, AMPK–Sirt3 pathway, Stress-induced hypertension

## Abstract

**Background:**

Neuroinflammation in the rostral ventrolateral medulla (RVLM) has been associated with the pathogenesis of stress-induced hypertension (SIH). Neuronal mitochondrial dysfunction is involved in many pathological and physiological processes. However, the impact of neuroinflammation on neuronal mitochondrial homeostasis and the involved signaling pathway in the RVLM during SIH are largely unknown.

**Methods:**

The morphology and phenotype of microglia and the neuronal mitochondrial injury in vivo were analyzed by immunofluorescence, Western blot, RT-qPCR, transmission electron microscopy, and kit detection. The underlying mechanisms of microglia-derived tumor necrosis factor‐α (TNF-α) on neuronal mitochondrial function were investigated through in vitro and in vivo experiments such as immunofluorescence and Western blot. The effect of TNF-α on blood pressure (BP) regulation was determined in vivo via intra-RVLM microinjection of TNF-α receptor antagonist R7050.

**Results:**

The results demonstrated that BP, heart rate (HR), renal sympathetic nerve activity (RSNA), plasma norepinephrine (NE), and electroencephalogram (EEG) power increased in SIH rats. Furthermore, the branching complexity of microglia in the RVLM of SIH rats decreased and polarized into M1 phenotype, accompanied by upregulation of TNF‐α. Increased neuronal mitochondria injury was observed in the RVLM of SIH rats. Mechanistically, Sirtuin 3 (Sirt3) and p-AMPK expression were markedly downregulated in both SIH rats and TNF-α–treated N2a cells. AMPK activator A769662 upregulated AMPK–Sirt3 signaling pathway and consequently reversed TNF-α–induced mitochondrial dysfunction. Microinjection of TNF-α receptor antagonist R7050 into the RVLM of SIH rats significantly inhibited the biological activities of TNF-α, increased p‐AMPK and Sirt3 levels, and alleviated neuronal mitochondrial injury, thereby reducing c-FOS expression, RSNA, plasma NE, and BP.

**Conclusions:**

This study revealed that microglia-derived TNF-α in the RVLM impairs neuronal mitochondrial function in SIH possibly through inhibiting the AMPK–Sirt3 pathway. Therefore, microglia-derived TNF-α in the RVLM may be a possible therapeutic target for the intervention of SIH.

**Supplementary Information:**

The online version contains supplementary material available at 10.1186/s12974-023-02818-6.

## Introduction

Hypertension, which is the largest single contributor to the global burden of disease, has attracted great attention worldwide [1]. A recent report predicts that approximately 1.5 billion adults suffer from hypertension by 2025 [1, 2]. Meanwhile, its high prevalence increases the health risk of various noncommunicable diseases, such as stroke, kidney damage, blindness, and congestive heart failure [3, 4]. The etiology of hypertension is extremely complex, involving not only genetic and behavioral factors, but also psychosocial factors [2]. The persistence of psychosocial stress contributes to the increased risk of hypertension, such as long periods working in a high-stress environment and relationship stress [5]. Stress-induced hypertension (SIH), whatever the cause, is associated with elevated sympathetic activity [6].

The rostral ventrolateral medulla (RVLM) is the key central region that refers to the integration of vasomotor input and contains presympathetic neurons responsible for the regulation of sympathetic output [7–9]. The activity of RVLM neurons is influenced by changes in the neurochemical milieu and plays a critical role in cardiovascular regulation [10–12]. Mounting evidence indicates that central neuroinflammation can excite the activity of neurons and further increases the sympathetic outflow, which is connected to the development of neurogenic hypertension [13–15]. Importantly, our previous study demonstrated neuroinflammation in the RVLM aggravated SIH development [16]. Stress-induced σ-1R inactivation upregulates microglial neuroinflammation in the RVLM, thereby resulting in sympathetic hyperactivity in SIH [17]. Mitochondria are essential organelles that maintain the normal physiological function of neurons through regulating pathways of cell metabolism and viability [18]. Mitochondrial dysfunction in neurons may play a major role in many disease processes. Fernandes et al*.* summarized that mitochondrial homeostasis impairment exacerbated neuronal proteotoxicity in the diabetic aging brain [19]. Jia et al*.* revealed the role of mitochondrial dysfunction as a marker of neuronal death in ischemic stroke [20]. Mitochondrial injury and the resulting reactive oxygen species (ROS) have been proposed as pivotal roles in the etiology of neurodegenerative diseases including Alzheimer’s disease and Parkinson’s disease [21]. Several studies also suggested that impaired mitochondria in cardiovascular regulation nuclei augmented sympathetic vasomotor tone, which further promoted hypertension progression [22, 23]. Our previous study indicated mitochondria damage may be linked with the pathogenesis of SIH [16]. However, whether microglial neuroinflammation is involved in SIH by mediating RVLM neuronal mitochondrial dysfunction remains unclear.

Sirtuin-3 (Sirt3), the deacetylase mainly located in mitochondria, participates in the regulation of mitochondrial functions, energy metabolism, and antioxidative defense systems [24, 25]. Cumulative evidence has shown that Sirt3 plays a crucial role in the occurrence and development of neurological diseases [26]. Lee et al*.* described that Sirt3 deregulation contributes to mitochondrial dysfunction and neuronal damage, which is associated with the pathogenesis of Alzheimer's disease [27]. Zhang et al*.* proposed that neuron-specific knockout of Sirt3 reversed the protective effects of adiponectin receptor agonist on the mitochondria after traumatic brain injury [28]. Sirt3 overexpression has been proven to improve mitochondrial impairment, inhibit oxidative stress, and further alleviate neuropathic pain [29]. In the current study, the Sirt3 expression was markedly decreased in the RVLM of SIH rats. However, whether Sirt3 dysregulation is implicated in the development of SIH and the relationship between Sirt3 and neuronal mitochondrial injury in the RVLM under microglial neuroinflammation are unknown.

The current work aimed to investigate the mechanisms involved in the effect of microglia-mediated neuroinflammation on neuronal mitochondrial function in SIH. The results demonstrated that microglia-derived tumor necrosis factor‐α (TNF-α) disrupted neuronal mitochondrial homeostasis in the RVLM though inhibiting the AMPK–Sirt3 pathway, and TNF-α suppression rescued neuronal mitochondrial injury and alleviated neuronal excitation, sympathetic outflow, and blood pressure (BP). Thus, the present study has implied that microglia-derived TNF-α could be exploited as a potential target for the prevention or treatment of SIH.

## Materials and methods

### Animals

**Fig. 1:**
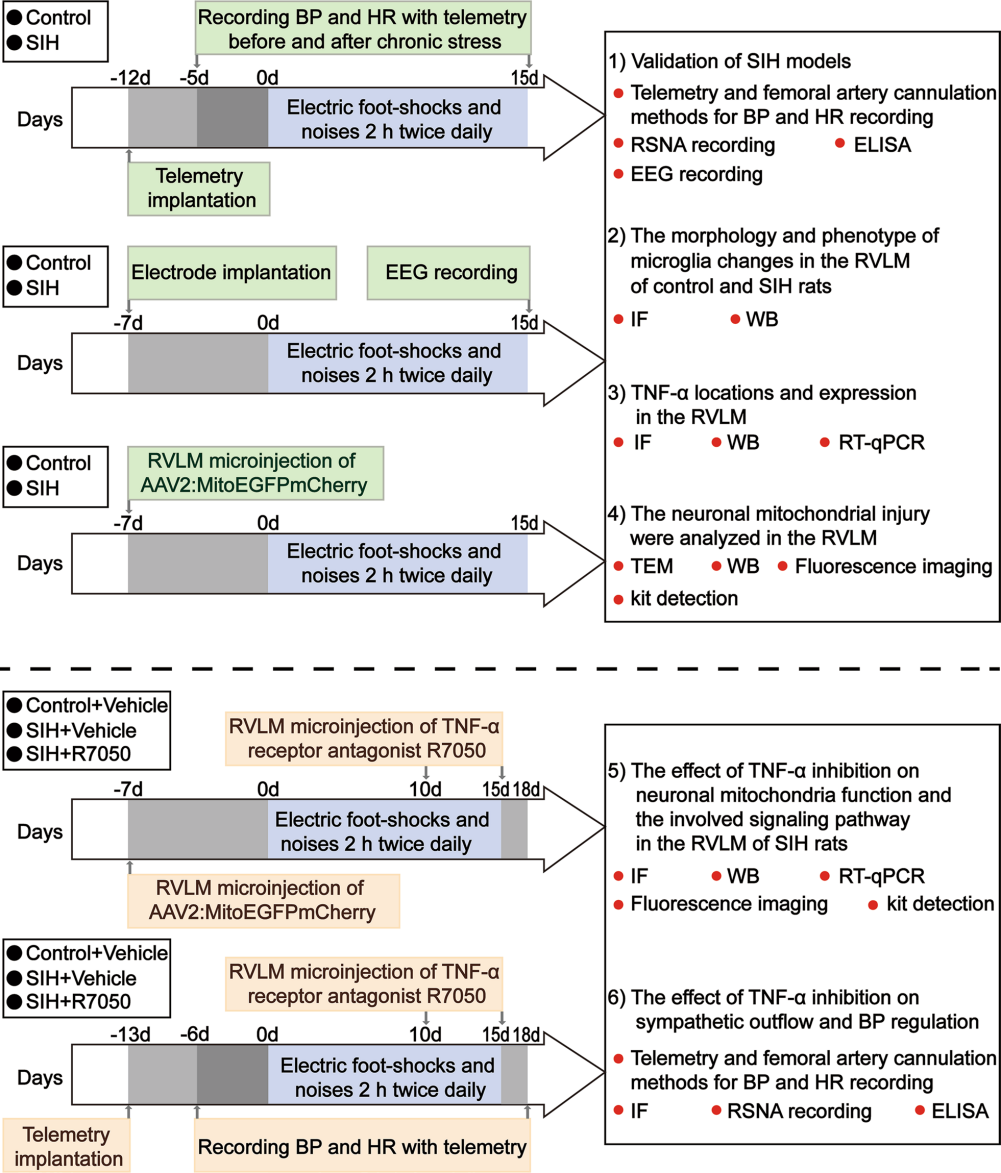
Animal experimental design. BP: blood pressure; EEG: electroencephalogram; ELISA: enzyme-linked immunosorbent assay; HR: heart rate; IF: immunofluorescence; RVLM: rostral ventrolateral medulla; RSNA: renal sympathetic nerve activity; RT-qPCR: reverse transcription quantitative polymerase chain reaction; SIH: stress-induced hypertension; TEM: transmission electron microscopy; TNF-α: tumor necrosis factor‐α; WB: Western blot

Adult male Sprague–Dawley rats (n = 220) weighing 250–300 g and aged eight weeks old were maintained in separate cages at a 12 h light/dark cycle condition at a room temperature (23 °C ± 1 °C) with the humidity around 50–60% in the specified pathogen-free facility of Shanghai University. The experimental animals were allowed unlimited access to food and tap water. All efforts were made to minimize the number of animals used and their suffering. Figure 1 showed the general study design. The SIH model was prepared as reported previously [6]. In the SIH group, rats were placed into a cage (22 cm × 22 cm × 28 cm) with a grid floor and suffered from electric foot shocks randomly controlled by a computer (35–80 V, 100 ms duration, 2–30 s intervals). Noises within the range of 88–98 dB produced by a buzzer was applied as a conditioned stimulus. The rats were subjected to stress for 2 h twice daily (9–11 a.m. and 3–5 p.m.) for 15 consecutive days. Rats in the control group underwent sham stress and placed into the same cages for the same time without foot shocks or noises. Our experimental procedures were in accordance with the Guidelines on the Care and Use of Laboratory Animals (National Institutes of Health Publication no. 85–23, revised 1996) and were approved by the Ethics Committee of Shanghai University. The ethics approval number is SYXK (HU) 2019–0020. No exclusion criteria were pre-determined. No randomization was performed to allocate subjects in the study.

### Cell culture and treatment

Mouse neuroblastoma N2a cell line was obtained from the Institute of Basic Medical Sciences of the China Science Academy. The cells were maintained in high glucose Dulbecco’s modified Eagle’s medium (DMEM, Cat.NO.10741574, Gibco, USA) supplemented with 10% fetal bovine serum (FBS, Cat.NO.11573397, Gibco, USA) in a humidified incubator with an atmosphere of 5% CO_2_/95% O_2_ at 37 °C. Mouse tumor necrosis factor‐α (TNF-α) was obtained from Novoprotein (Cat.NO.CF09, China). The AMPK activator A769662 was purchased from MedChemExpress (Cat.NO.HY-50662, USA). N2a cells were treated separately for 24 h with TNF-α (30 ng/ml) or TNF-α (30 ng/ml) plus A769662 (30 and 60 μM).

### Measurements of BP and heart rate (HR)

The BP and HR of conscious rats were continuously measured by the radiotelemetry system (TRM54P, Kaha Sciences, New Zealand), as reported previously [30, 31]. After the rats were anesthetized with inhaled isoflurane (oxygen flowmeter: 0.8–1.5 L/min, isoflurane vaporizer: 1.5–3%), the telemeter catheter was inserted into abdominal aorta and fixed with tissue adhesive and a mesh patch. The body of the telemeter was implanted into the abdominal cavity and secured to the peritoneum. Following telemeter implantation, animals were given 7 days to recover from the surgery. The BP and HR signals of the rats were received by SmartPads (TR180, Kaha Sciences, New Zealand), and the data were collected in a fixed period of 4 h (from 19 to 23 o’clock) every day. Levels of BP and HR of anesthetized rats were recorded by femoral artery cannulation using the PowerLab system (AD Instruments, Australia) [17]. Briefly, After the rats were anesthetized with inhaled isoflurane as above, the right femoral vein and artery were separated by blunt dissection. The polyethylene catheter of PE 50 tubing (with 0.01% heparinized saline to prevent clotting) was inserted into the right femoral artery. The distal end of the arterial cannula was attached to a pressure transducer for the recording of BP. HR was automatically derived from the phasic arterial BP wave by computer. During the experiment, the body temperature was continuously monitored by a rectal thermometer and maintained at 37 °C by using a temperature controller (H-KWDY-III, Quanshui Experimental Instrument, China).

### Renal sympathetic nerve activity (RSNA) recording

The rats were anesthetized with inhaled isoflurane as above and the left kidney, renal artery, and sympathetic nerves were exposed through a retroperitoneal incision [32]. The left renal sympathetic nerve was carefully isolated and placed on a pair of silver recording electrodes. Subsequently, the exposed nerve and the electrodes were covered with silicone gel (Kwik-Sil, WPI, USA) and a low-noise differential amplifier was used to amplify (×100) and filter (band-pass 100–3000 Hz) the nerve activity. RSNA signals were integrated and recorded by the PowerLab system (AD Instruments, Australia). The maximum activity was detected when rats were overdosed by narcotic euthanasia (pentobarbital: 200 mg/kg, i.p.). The background noise level was recorded 20–30 min after the rats were sacrificed. Baseline RSNA value was taken as a percentage of maximal RSNA after detracting the background noise.

### Enzyme-linked immunosorbent assay (ELISA)

Rat blood samples were obtained by cardiac puncture using EDTA-Na_2_ as an anticoagulant under anesthesia with isoflurane as above. The samples were centrifuged at 1000*g* for 15 min at 4 °C to isolate plasma. The plasma was collected for detection. The plasma norepinephrine (NE) concentration was measured by using NE ELISA kit (Cat.No.EU2565, FineTest, China) according to the manufacturer’s instructions. In brief, the standards and samples were added to a microplate pre-coated with the universal NE antigen, followed by the addition of biotin-conjugated anti-universal NE antibodies. After incubation, excess conjugates and unbound standards or samples were washed from the microplate, and the HRP-conjugated streptavidin was added into the wells. Next, the TMB substrate solution was added to each well after washing the microplate. The enzyme–substrate reaction was terminated by the addition of a sulphuric acid solution. Finally, the absorbance was assessed at 450 nm using a SpectraMax iD5 microplate reader (Molecular Devices, USA). The results were presented as picogram per milliliter.

### Electroencephalograph recording

For electrode implantation, rats were placed on the stereotaxic apparatus (69100, RWD Life Science, China) under anesthesia with isoflurane as the above mentioned. Dental cement was used to embed electrode bundles (Teflon-coated stainless-steel wires, 50 mm diameter, 5 channels) in the skull. These electrodes were implanted into the rostral ventrolateral medulla (RVLM) (3.7–4.0 mm caudal to lambdoid suture, 2 mm lateral to the midline, and 8.0 mm ventral to the surface of the dura) according to the atlas of Paxinos and Watson [33]. The electrophysiological signals, which were conducted in freely moving rats, were recorded using a Plexon multichannel acquisition system. The signals were initially band-pass filtered at 0.5–200 Hz with a second-order Butterworth filter, further amplified, and digitized at 1 kHz by the Plexon multichannel acquisition system. Matlab R2019a (Mathworks, USA) was used to analyze the electrophysiological signals. Ten seconds of data were removed from the beginning of every trial to reduce edge artifacts. The analysis of power spectral density (PSD) was performed using Welch formula in a period of 200 s in each sample. The power of δ (1–4 Hz), θ (4–8 Hz), α (8–15 Hz), β (15–30 Hz), low γ (30–60 Hz), and high γ (60–100 Hz) rhythm in electroencephalogram (EEG) was analyzed for each electrode in every animal. The data were calculated by averaging across each electrode for an animal, and then averaging across each animal for every group.

### Immunofluorescence

Immunofluorescence staining was performed as described previously [34]. The rats were anesthetized with pentobarbital sodium (50 mg/kg, i.p.) and perfused through the left ventricle with 200 ml of heparinized saline, followed by 200 ml of freshly prepared 4% paraformaldehyde in 0.1 M PBS (pH7.4). Brain tissues were removed, post-fixed for 12 h, and placed in 20% sucrose at 4 °C until the brains sunk to the bottom, and then in 30% sucrose at 4 °C until the brains sunk to the bottom. Frozen coronal sections (30 μm) containing the RVLM were sliced using a cryostat (HM525, Microm, Germany) and preserved in situ in hybridization protection fluid. The sections were washed three times for 5 min in PBS and incubated with 0.3% Triton X-100 for 30 min, followed by incubation with 5% goat serum for 1 h at 37 °C to block nonspecific protein. The sections were incubated with the goat polyclonal Iba1 antibody (1:400, Cat.No.ab5076, Abcam, UK), rabbit monoclonal Iba1 antibody (1:400, Cat.No.ab178846, Abcam, UK), mouse monoclonal NeuN antibody (1:400, Cat.No.ab104224, Abcam, UK), rabbit monoclonal NeuN antibody (1:400, Cat.No.ab177487, Abcam, UK), rabbit polyclonal GFAP antibody (1:400, Cat.No.ab7260, Abcam, UK), mouse monoclonal TNF-α antibody (1:50, Cat.No.sc-52746, Santa Cruz, USA), rabbit monoclonal Sirt3 antibody (1:200, Cat.No.#2627, Cell Signaling Technology, USA), mouse monoclonal TH antibody (1:100, Cat.No.sc-25269, Santa Cruz, USA), and rabbit monoclonal c-FOS (1:1000, Cat.No.#2250, Cell Signaling Technology, USA) overnight at 4 °C. The following day, sections were washed with PBS and incubated with secondary antibodies, Alexa Fluor 674 conjugated with goat anti-mouse IgG (1:600, Cat.No.ab150115, Abcam, USA), Alexa Fluor 647 conjugated with donkey anti-goat IgG (1:600, Cat.No.ab150135, Abcam, USA), Alexa Fluor 594 conjugated with goat anti-rabbit IgG (H + L) (1:400, Cat.No.111-585-003, Jackson ImmunoResearch, USA), and FITC conjugated with goat anti-mouse IgG (H + L) (1:200, Cat.No.115-095-003, Jackson ImmunoResearch, USA) for 2 h at room temperature. The fluorescent signals were monitored under a confocal laser scanning microscope (LSM880, ZEISS, Germany); immunoreactivity manifested as specific green or red fluorescence.

### Image quantification procedures

Confocal Z-stack images were taken using a confocal laser scanning microscope (LSM880, ZEISS, Germany) at × 40 or × 63 magnification consisting of at least 12 images. Images of microglia were subjected to maximum intensity projection, and only cells whose branches were completely within the 3D Z-stack volume were quantified. We performed skeletonization analysis and Sholl analysis to quantify the morphological changes of microglia using Image J software. For mCherry-ONLY volume comparisons, the confocal images were transformed into 3D reconstruction files using Imaris 9.7 software. Numbers of EGFP puncta and mCherry puncta were determined using the spots module in Imaris 9.7. The mCherry-ONLY signal was quantified by zeroing the number in EGFP puncta and counting the remaining mCherry puncta. Other images were processed and analyzed using the Image J software.

### Cytosol fraction isolation

The cytosol fraction was isolated using the mitochondria isolation kit (Cat.No.abs9344, absin, China) according to the manufacturer’s protocol. Briefly, the RVLM tissue was washed and homogenized using pre-cooled lysis buffer, and then was centrifuged at 600*g* for 5 min at 4 °C. The sediment was removed and the supernatant was transferred to another tube and further centrifuged at 12,000*g* for 10 min at 4 °C. Finally, the collected supernatant was used for Western blot analysis.

### Western blot analysis

Total or cytosol protein extracted from RVLM tissue and total protein extracted from N2a cells were used to analyze protein expression by Western blot. Proteins were separated by SDS-PAGE in 4–12% gradient gel and transferred into the PVDF membranes (Millipore, Cat.No.IPVH00010, USA). The membranes were blocked with QuickBlock™ Western (Beyotime, Cat.No.P0252, China) for 1 h prior to incubation at 4 °C overnight with primary antibodies including rabbit monoclonal antibody against iNOS (1:1000, Cat.No.ab178945, Abcom, USA), rabbit monoclonal antibody against CD86 (1:1000, Cat.No.a19026, ABclonal, China), rabbit monoclonal antibody against Arg-1 (1:1000, Cat.No.A4923, ABclonal, China), mouse monoclonal antibody against TNF-α (1:1000, Cat.No.sc-52746, Santa Cruz, USA), rabbit polyclonal antibody against p-AMPK (1:500, Cat.No.AF5908, Beyotime, China), rabbit monoclonal antibody against AMPK (1:1000, Cat.No.AF1627, Beyotime, China), rabbit monoclonal antibody against Sirt3 (1:1000, Cat.No.#2627, Cell Signaling Technology, USA), rabbit polyclonal antibody against ATP5A (1:1500, Cat.No.GB113455, Servicebio, China), mouse monoclonal antibody against UQCRC2 (1:1000, Cat.No.sc-390378, Santa Cruz, USA), rabbit monoclonal antibody against SDHB (1:1000, Cat.No.AG3213, Beyotime, China), mouse monoclonal antibody against TNFR1 (1:500, Cat.No.sc-8436, Santa Cruz, USA), mouse monoclonal antibody against TNFR2 (1:500, Cat.No.sc-12750, Santa Cruz, USA), and mouse monoclonal HRP-Conjugated β-actin antibody (1:5000, Cat No.HRP-60008, Proteintech, USA). After being washed with PBS and Tween 20 (PBST) buffer, the membranes were then incubated at goat anti-rabbit IgG (1:5000, Cat.NO.ab205718, Abcam, USA) or goat anti-mouse IgG (1:5000, Cat.No.ab205719, Abcam, USA) for 1 h at room temperature. The amount of protein was assessed using super ECL detection reagents (Cat.No.RM00021, Abclonal, China), and the immunostaining band was detected by an automatic chemiluminescence image analysis system (Tanon-5200, Tanon Science & Technology, China). The data were normalized by developing the β-actin as loading control. Following the initial detection, as needed, we washed the targeted band protein by restore™ western blot stripping (Cat.No.21059, Thermo Scientific, USA) for 30–60 min to remove the previous antibody. Subsequently, the PVDF membranes were reblocked for 1 h by QuickBlock™ Western after being washed three times with PBS. Finally, those membranes were incubated at 4 °C overnight with other primary antibodies. In this study, the membranes are reused at most once.

### Total RNA extraction and reverse transcription quantitative polymerase chain reaction (RT-qPCR)

Total RNA from the RVLM or cell was extracted with TRIzol^®^ reagent (Cat.No.15596026, Invitrogen, USA) and converted into first-strand cDNA (Cat.No.11119ES60, Yeasen, China) in accordance with the manufacturer’s instructions. Quantitative real-time PCR was performed using the Hieff^®^ qPCR SYBR Green Master Mix (Cat.No.11195ES03, Yeasen, China). Amplification and melting curves were recorded using a CFX96 Touch Real-time PCR Detection System. The sequences of primers are listed in Table 1**.** The relative quantification of gene expression was expressed as fold change via normalization against GAPDH.

**Table 1:** Primers used for reverse transcription quantitative polymerase chain reaction (RT-qPCR) analysis

Gene	Primer (5′–3′)	Annealing temperature (°C)	The size of production (bp)
TNF-α	F: CCTCACCCACACCGTCAG R: GCAGGTCCCCCTTCTCCA	60 °C	170 bp
TNFR1 TNFR2	F: ATGTCGGAAAGAAATGTTC R: ATGCGTCTCACTCAGGTAG F: TAGGACTGGCGAACTGCT R: GACTCTTGCTTGGGGATG	60 °C 60 °C	109 bp 230 bp
GAPDH	F: GTCGGTGTGAACGGATTTG R: TCCCATTCTCAGCCTTGAC	60 °C	181 bp

### Transmission electron microscopy (TEM)

The sample preparation for TEM was performed the same as in our previous publication [16]. The RVLM was sliced into 1-mm transverse sections and fixed in 4% paraformaldehyde and 1% glutaraldehyde overnight. The sections were subsequently postfixed in 1% phosphate-buffered osmium tetroxide (2 h), dehydrated in the graded concentration ethanol, and carefully embedded in epoxy resin. The 60–70 nm ultrathin sections obtained from the ultramicrotome were post-stained with uranyl acetate and lead citrate and examined using a TEM (HT7800, Hitachi, Japan).

### Intra-RVLM microinjection

Intra-RVLM microinjection was performed as described in our previous study [35]. Briefly, the rats were anesthetized with inhaled isoflurane as above and placed in the prone position, and their heads were fixed in the stereotaxic apparatus (69100, RWD Life Science, China). The skull was well exposed by a midline incision. The lambda and bregma skull points were located on the same horizontal plane. As stated in the previous literature [36], the MitoEGFPmCherry construct was made in a pCS2 + vector by in-frame fusion of amino acids 1–29 of human (and other primate) cytochrome c subunit 8 to EGFP, and then by a flexible glycine linker, followed by mCherry. An adeno-associated virus (AAVs) containing MitoEGFPmCherry production and purification was carried out at Hanbio (China). R7050 (TNF-α receptor antagonist) was purchased from MedChemExpress (Cat.NO.HY-110203, USA) and was dissolved in Vehicle (NaCl, 0.9%) at a dosage of 10 µM. The infusion dose was based on previous studies [36, 37]. According to the atlas of Paxinos and Watson [33], AAV2:MitoEGFPmCherry or R7050 was bilateral microinjected into the RVLM (coordinates: 3.7–4.0 mm caudal to lambdoid suture, 2 mm lateral to the midline, and 8.0 mm ventral to the surface of the dura) at 0.5 μL/side by a glass micropipette. We have described the schematic diagram to prove the process of determining the correct positioning of RVLM in the previous literature [35]. The time points of the microinjection are exhibited in Fig. 1. The incision was well cleaned and sutured after the microinjection was completed.

### ROS assay

According to previous study [38], the ROS production in the RVLM was detected by dihydroethidium (DHE, Cat.No.S0063, Beyotime, China). Frozen brains sliced into 30 µm sections on a cryostat (HM525, Microm, Germany). The brain sections were incubated with 5 μM DHE for 30 min at 37 °C and then washed three times with 0.1 M PBS. The fluorescence was detected by a confocal laser scanning microscope (LSM880, ZEISS, Germany). Intracellular ROS production in N2a cells were measured by a ROS assay kit (Cat.No.S0033M-1, Beyotime, China) per manufacturer’s instructions. Experiments were performed in 12 wells, in which cells were incubated with 10 μM DCFH-DA at 37 °C for 20 min in a dark room. Then, they were washed three times with serum-free medium, and the signals were detected under a confocal laser scanning microscope (LSM880, ZEISS, Germany). The level of ROS was evaluated by the relative fluorescence intensity of each experimental group with Image J software.

### Determination of superoxide dismutase (SOD) and catalase (CAT) activity

The SOD and CAT activity levels in the RVLM and cultured N2a cells were determined using commercially available SOD and CAT kits (Cat.No.S0103, Cat.No.S0051, Beyotime, China) following the manufacturer’s instructions. The data were analyzed spectrophotometrically using a SpectraMax iD5 microplate reader (Molecular Devices, USA).

### Cell counting kit-8 (CCK-8) assay

The cell viabilities of N2a cells after TNF-α or TNF-α plus A769662 (AMPK Activator) treatment were estimated using CCK-8 assay kit (Cat.No.B34304, Bimake, USA). N2a cells (1 × 10^4^ cells/well) were seeded into 96-well plates and cultured for 24 h followed by 24 h incubation with TNF-α (30 ng/ml) or TNF-α (30 ng/ml) plus A769662 (30 and 60 μM). Then, the medium was replaced with fresh medium. The CCK-8 working solution (10 μl) was added into each well and incubated together for 30 min. Optical density was measured at 450 nm using a SpectraMax iD5 microplate reader (Molecular Devices, USA).

### Measurement of the mitochondrial membrane potential (MMP)

MMP were evaluated with a commercial kit with JC-1 staining (Cat.No.C2006, Beyotime, China). The N2a cells in different groups were covered with 500 µl JC-1 working solution and incubated at 37 ℃ for 20 min for full staining, and then washed with dyeing buffer twice. The fluorescence level was measured immediately using a laser scanning confocal microscope (LSM880, ZEISS, Germany), where JC-1 aggregated in normal mitochondria to form polymers and emit red fluorescence, whereas unhealthy mitochondria can only exist in monomer form and produce green fluorescence due to the decrease or loss of membrane potential. The degree of mitochondrial depolarization can be detected by the ratio value of red to green fluorescence intensity.

### Statistical analysis

Statistical analysis was performed using v 9.1 Graphpad Prism software. Data are presented as means ± standard error of the mean (SEM). The normality of the data was evaluated by the Shapiro–Wilk test. The data conformed to a normal distribution. Student’s unpaired *t* test was used for experiments that contained two groups of samples. For comparison of multiple groups, one-way ANOVA with subsequent post hoc Bonferroni test was conducted. Sholl analysis data were analyzed by two-way repeated measures ANOVA.* P* < 0.05 indicated that the differences were statistically significant.

## Results

### BP, HR, RSNA, NE, and electroencephalogram (EEG) power were increased in SIH rats

**Fig. 2:**
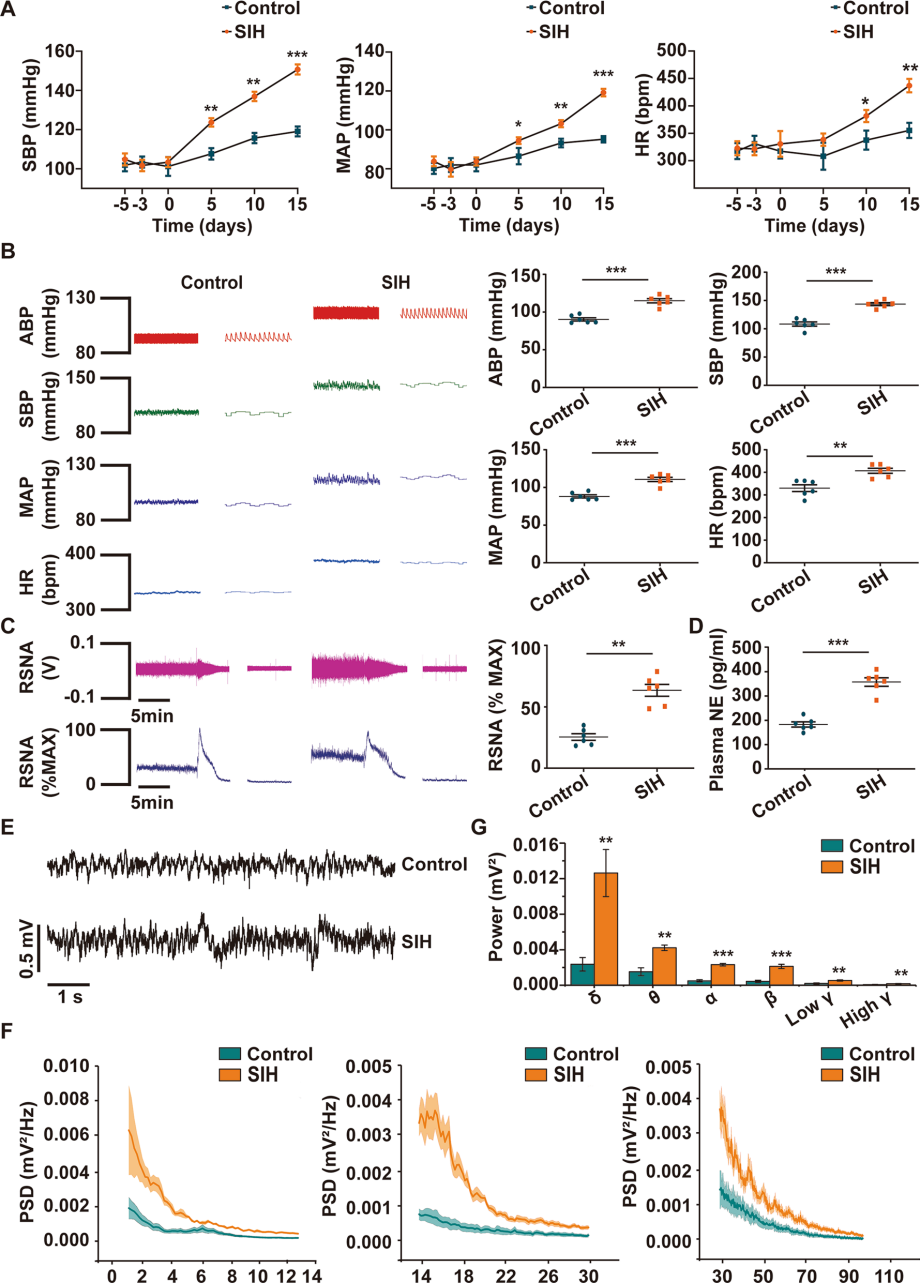
Changes in blood pressure (BP), heart rate (HR), renal sympathetic nerve activity (RSNA), plasma norepinephrine (NE), and electroencephalogram (EEG) power between control and SIH rats. **A** The level of systolic blood pressure (SBP), mean arterial pressure (MAP), and HR before and after chronic stress in conscious rats. **B** The arterial blood pressure (ABP), SBP, MAP, and HR were tested in anesthetized rats. **C** Renal sympathetic nerve activity (RSNA) recording and **D** plasma norepinephrine (NE) ELISA assay were performed in the two groups. **E** Representative traces of EEG recording in the rostral ventrolateral medulla (RVLM) of control and SIH rats. **F** Power spectral density (PSD) features of control and SIH rats and **G** statistical data for power among δ, θ, α, β, Low γ, and High γ. Data were shown as mean ± SEM and analyzed via two-tailed unpaired Student’s *t*-test (**A**–**G**). n = 6 rats per group (**A**–**G**). *p < 0.05, **p < 0.01, ***p < 0.001 vs. Control group

Rats were subjected to electric foot shock stressors with buzzer noises for 2 h twice per day for successive 15 days. The results of hemodynamic parameters of rats under conscious and anesthetized conditions were shown in Fig. 2. In conscious rats, the BP and HR of rats were recorded everyday using radiotelemetry system in conscious state after chronic stress. We observed that the systolic blood pressure (SBP), mean arterial pressure (MAP) and HR increased in a time-dependent manner in SIH rats (Fig. 2A). In anesthetized rats, after chronic stress for 15 days, the increases in arterial blood pressure (ABP), SBP, MAP, and HR were found in the SIH group compared with the control group (Fig. 2B). The RSNA and the level of plasma NE were also elevated in the SIH group (Fig. 2C, D). Subsequently, the EEG recording was performed to detect RVLM neuronal activity of rats (Fig. 2E). The PSD analysis showed that the power of EEG in SIH rats was greater than that in control rats (Fig. 2F). Further statistical analysis revealed that the power in all frequency bands was higher in the SIH group than in the control group (Fig. 2G): 1–4 Hz (delta, δ), 4–8 Hz (theta, θ), 8–15 Hz (alpha, α), 15–30 (beta, β), 30–60 Hz (Low gamma, Low γ), and 60–100 Hz (High gamma, High γ). These results indicated that chronic stress had a significant effect on BP, HR, sympathetic activity, and RVLM neuronal excitability of rats. Therefore, the rat model of SIH was reliable and accurately supported by these data.

### Microglia polarized to a M1 proinflammatory phenotype, accompanied by increased level of TNF-α in the RVLM of SIH rats

**Fig. 3:**
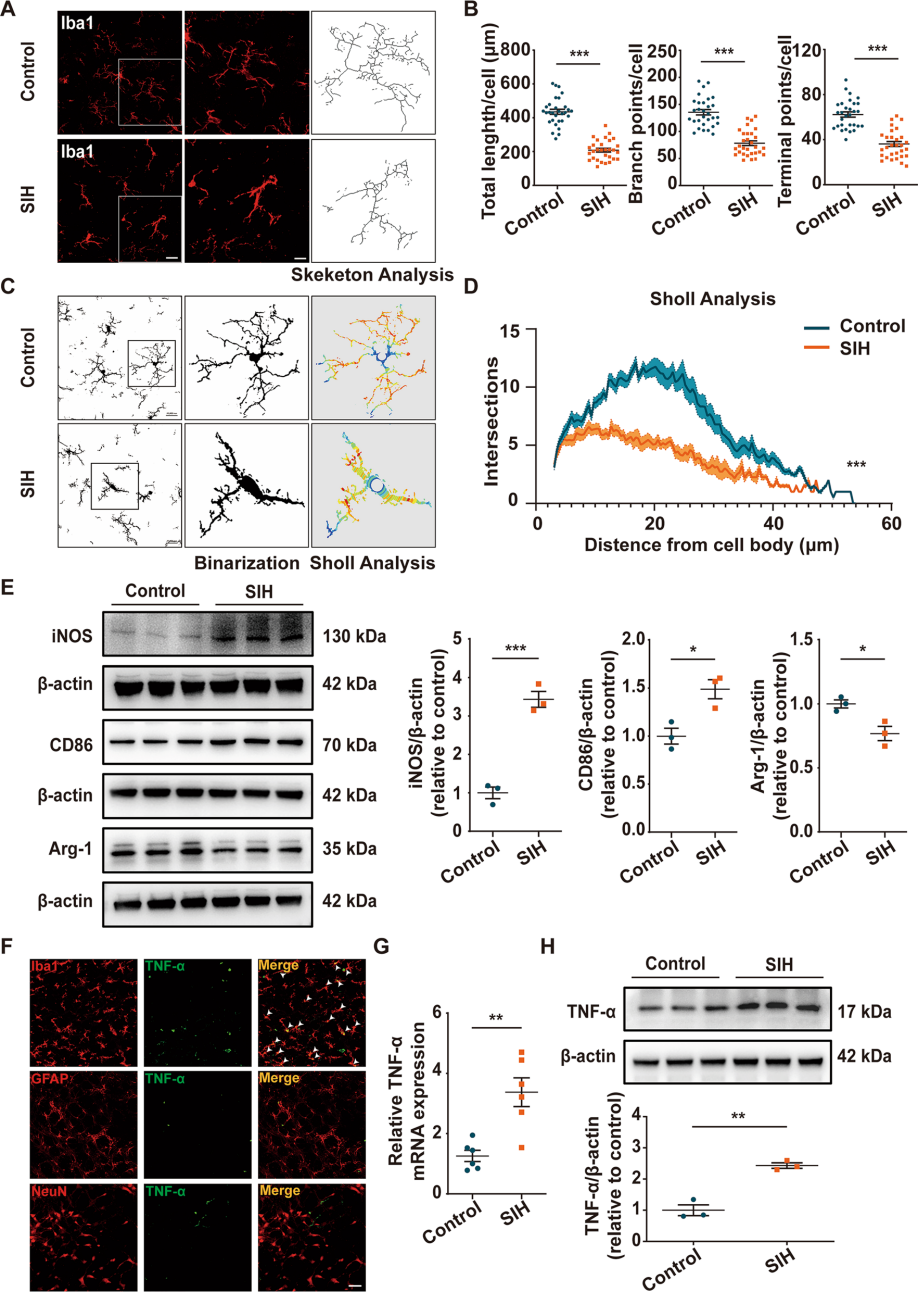
Microglia polarized into the proinflammatory M1 type, accompanied by an increase in TNF‐α in the RVLM of SIH rats. **A** Representative images of Iba1 immunofluorescence and the Z-stack projection of microglia in the RVLM of control and SIH rats. Scale bar = 20 or 5 μm. **B** Skeletal microglia analysis of total process length, the number of branching points, and the number of terminal points in control and SIH rats. **C**,** D** Sholl analysis of RVLM microglial processes in control and SIH rats. **E** Representative immunoblot bands and quantitative analysis showed the expression of iNOS, CD86, and Arg-1 in the RVLM between the two groups. **F** Immunofluorescence co-localization analysis of TNF-α in the RVLM. Iba1, GFAP, and NeuN were used as markers for microglia, astrocytes, and neurons, respectively. Scale bar = 50 μm. **G**, **H** The TNF-α expression was determined by RT-qPCR and Western blot assays in the RVLM of control and SIH rats. Data were expressed as mean ± SEM. Statistical significance was determined using two-tailed unpaired Student’s *t*-test (**A**,** B**,** E**, **G**, **H**) and two-way repeated measures ANOVA (**C**,** D**). n = 30 microglia from 6 rats (**A–D**). n = 3 rats per group (**E**, **H**). n = 6 rats per group (**G**). *p < 0.05,  **p < 0.01, ***p < 0.001 vs. Control group

Activated microglia, exhibiting morphology changes, can be polarized to M1 or M2 phenotype [39]. We initially compared the morphology of microglia in the RVLM of SIH rats and controls. The microglial morphology was measured using the confocal Z-stack projections (Fig. 3A). Skeletal microglia analysis showed that the total length of microglial branches in SIH group was less than that in control group, and the number of branches and the endings per cell were also reduced (Fig. 3B). Sholl analysis further revealed that the microglial process complexity of SIH group was decreased (Fig. 3C, D). Subsequently, Western blot was performed to examine the phenotype of microglial activation in the RVLM. The results revealed that the expression of iNOS and CD86 in SIH rats was higher, whereas that of Arg-1 was lower than that of control rats, suggesting that microglia polarized to the M1 proinflammatory phenotype (Fig. 3E). TNF-α is well recognized pro-inflammatory cytokine, which can trigger an augmented inflammatory response; it affects many different biological processes and diseases [40, 41]. The immunofluorescence co-localization analysis showed TNF-α was mainly localized in Iba1 (microglia marker)-positive cells but rarely in NeuN (neuron marker)-positive cells and GFAP (astrocyte marker)-positive cells in the RVIM of rats (Fig. 3F). Furthermore, RT-qPCR and Western blot results showed that the level of TNF-α was significantly elevated in the RVLM of SIH group compared to the control group (Fig. 3G, H). These results showed that microglia underwent morphologic changes and polarized into M1 proinflammatory phenotype, accompanied by upregulation of TNF‐α in the RVLM of SIH rats.

### Mitochondria were injured in the RVLM neurons of SIH rats

**Fig. 4:**
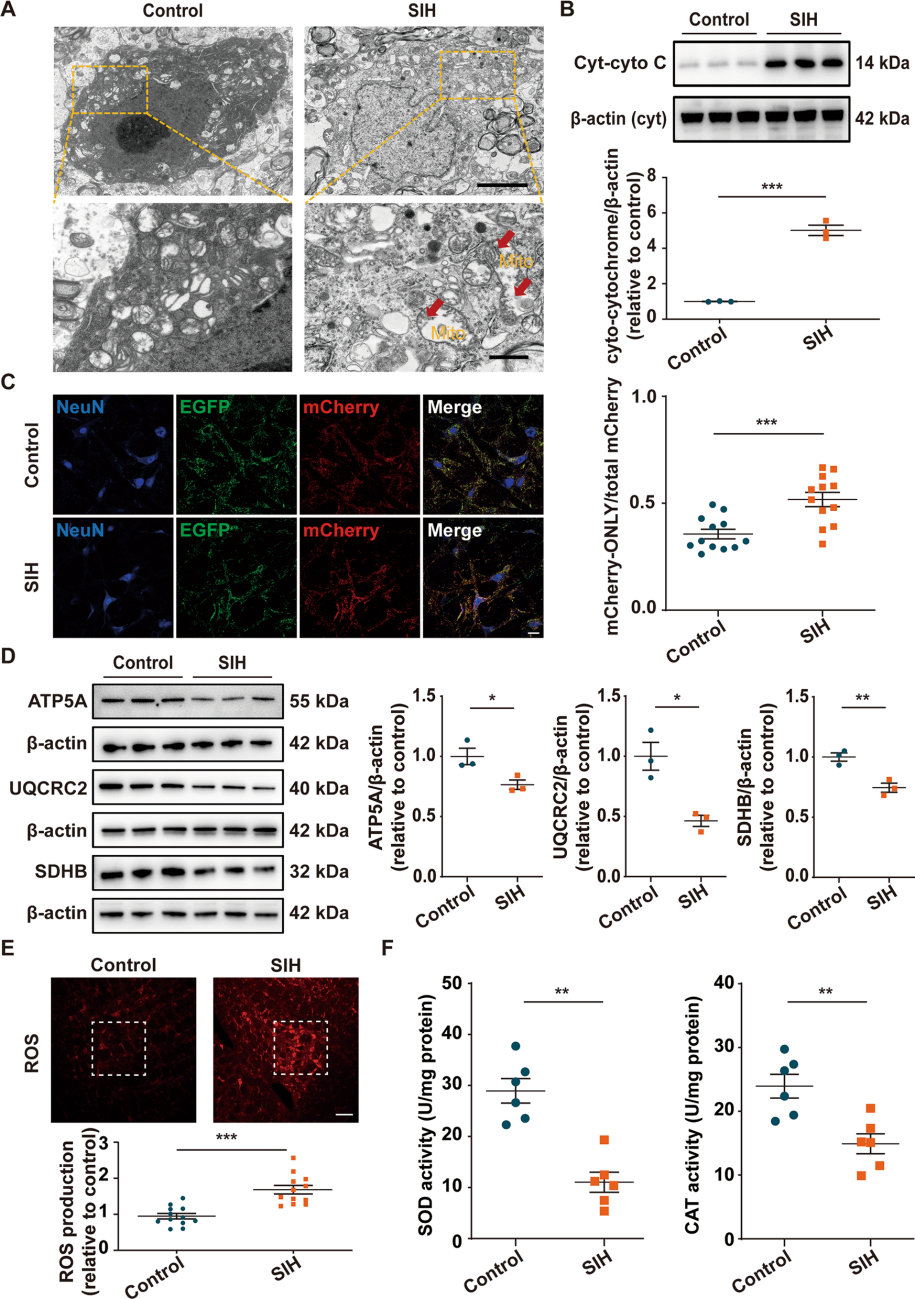
RVLM neuronal mitochondria were injured in SIH rats. **A** Representative photomicrographs of the RVLM neurons in control and SIH rats for visualization in transmission electron microscopy. Mito, mitochondria. Red arrows indicate disruption of the mitochondria with abnormal mitochondrial crests. Scale bar = 5 or 1 μm. **B** Representative immunoblot bands and quantitative analysis showed the expression of cytoplasmic cytochrome C (Cyt-cyto C) in the RVLM between the two groups. **C** Within the RVLM, AAV2:MitoEGFPmCherry infection produced punctate labeling of EGFP and mCherry, which colocalized with neuron marker NeuN. Scale bar = 20 μm. Quantitative analysis showed the degree of mitochondrial degradation by assessing the ratio value of mCherry-ONLY/total mCherry puncta. **D** Representative immunoblot bands and quantitative analysis showed the expression of mitochondrial respiratory chain complexes (complexes II-SDHB, III-UQCRC2, and V-ATP5A) in the RVLM of control and SIH rats. **E** Representative fluorescence images of reactive oxygen species (ROS) detection and statistical data of ROS production in the RVLM between the two groups. Scale bar = 100 μm. **F** The levels of superoxide dismutase (SOD) and catalase (CAT) were quantified by using commercial kits in each group. Data were shown as mean ± SEM and analyzed via two-tailed unpaired Student’s *t*-test (**B**–**F**). n = 3 rats per group (**B**, **D**). n = 6 rats per group (**F**). n = 12 slices from 6 rats, two slices per rat (**C**, **E**). *p < 0.05, **p < 0.01, ***p < 0.001 vs. Control group

As shown in Fig. 4A, compared with the control rats, the mitochondria in the RVLM neurons of SIH rats showed ultrastructural alterations containing excessive elongation, swelling, and blurred crista contours at the electron microscopy level. Subsequently, we evaluated the integrity of mitochondria by monitoring the protein level of cytoplasmic cytochrome C (Cyt-cyto C). Cytochrome C is a vital component of the electron transport chain in mitochondria. Once the integrity of mitochondria is impaired, the expression of Cyt-cyto C would be significantly upregulated [42]. The results showed that SIH rats had elevated levels of Cyt-cyto C, suggesting that the integrity of mitochondria was markedly impaired in the RVLM of SIH rat (Fig. 4B). The mitochondrial injury is degraded within lysosomes, and a tandem fluorophore reporter containing the acid-sensitive fluorophore EGFP and the acid-resistant fluorophore mCherry was designed to track mitochondria degradation [36]. The mCherry-positive/EGFP-negative (mCherry-ONLY) puncta can be used to identify degrading mitochondria. Here, the tandem fluorophore reporter of EGFP and mCherry was expressed in the RVLM by microinjecion of an adenoassociated virus 2 (AAV2) vector. Most of the EGFP and mCherry fluorescence colocalized with the neuron marker NeuN, confirming that the MitoEGFPmCherry reporter can be targeted to RVLM neuronal mitochondria (Fig. 4C). Using Imaris to compare the amount of the mCherry-ONLY puncta in the RVLM neurons revealed that the signal in the SIH group was significantly higher than in the control group (Fig. 4C). The protein levels of mitochondrial respiratory chain complexes (complex II-SDHB, complex III-UQCRC2, and complex V-ATP5A) decreased significantly in the RVLM of SIH rats, indicating that mitochondrial respiratory metabolism was impaired (Fig. 4D). Since mitochondria are the main intracellular sources of ROS [43], we measured ROS production in the RVLM to evaluated mitochondrial oxidative stress of SIH rats. As shown in Fig. 4E, the ROS levels in the SIH group were significantly higher than those in the control group. Moreover, relative to the control rats, the SIH rats exhibited markedly decreased superoxide dismutase (SOD) and catalase (CAT) activities (Fig. 4F). These results implied that neuronal mitochondria were evidently injured in the RVLM of SIH rats.

### TNF-α mediated mitochondrial dysfunction in neurons by inhibiting the AMPK-Sirt3 pathway

**Fig. 5:**
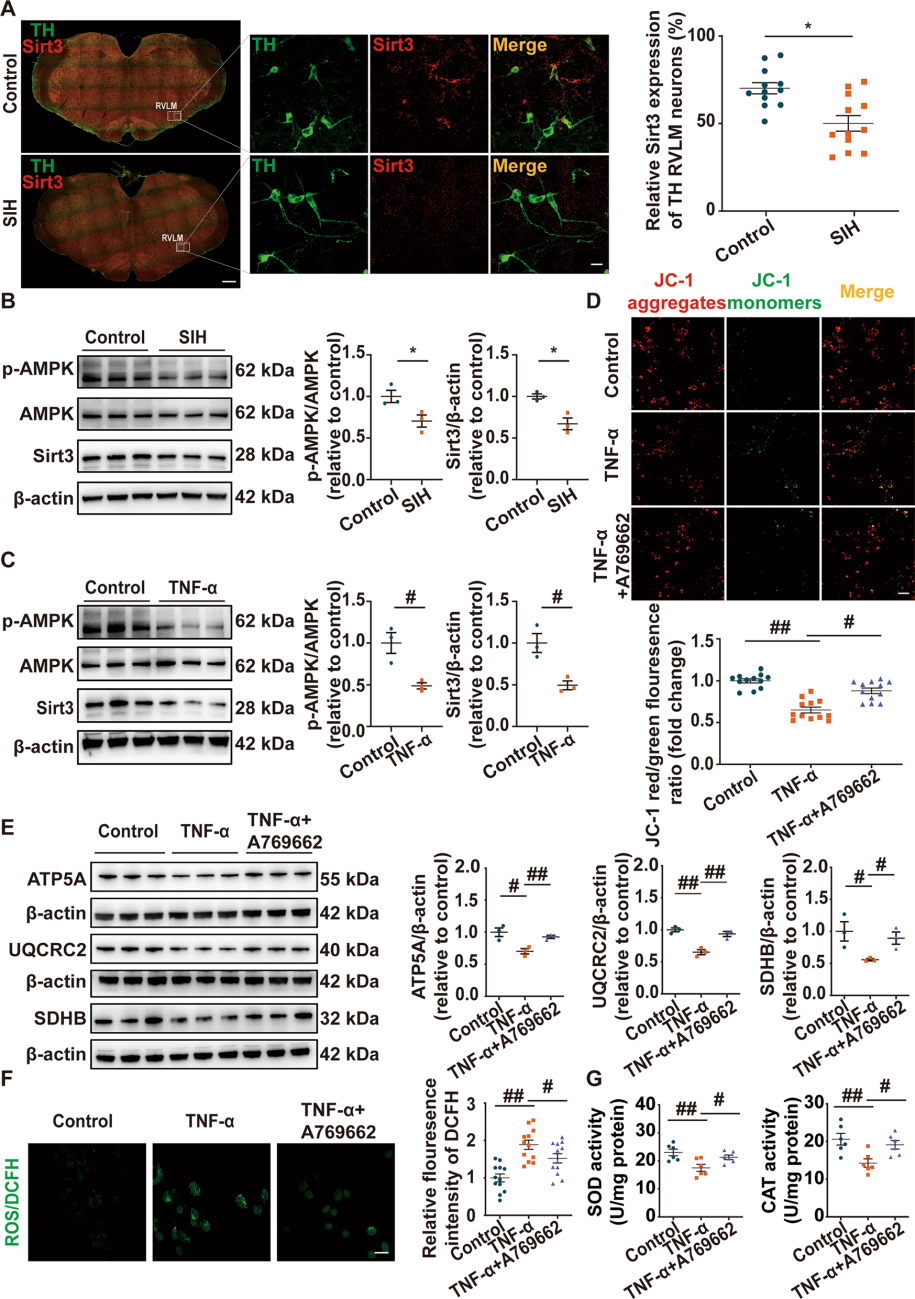
TNF-α led to neuronal mitochondrial dysfunction via downregulating the AMPK-Sirt3 pathway. **A** Representative immunofluorescence images and quantitative analysis showed colocalization of Sirt3 and tyrosine hydroxylase (TH)-positive neurons in the RVLM of control and SIH groups. Scale bar = 500 or 20 μm. **B** Representative immunoblot bands and quantitative analysis showed the expression of p-AMPK and Sirt3 in the RVLM of control and SIH rats. **C** Representative immunoblot bands and quantitative analysis of p-AMPK and Sirt3 expression in N2a cells treated with or without TNF-α. **D** Representative images and quantitative analysis showed mitochondrial membrane potential (MMP) in control, TNF-α, and TNF-α + A769662 groups. Scale bar = 50 μm. **E** Representative immunoblot bands and quantitative analysis showed the expression of mitochondrial respiratory chain complexes (complexes II-SDHB, III-UQCRC2, and V-ATP5A) in N2a cells with different treatments. **F** Representative fluorescence images of reactive oxygen species (ROS) detection and statistical data of ROS production in N2a cells with or without TNF-α treatment. Scale bar = 50 μm. **G** The levels of superoxide dismutase (SOD) and catalase (CAT) were quantified by using commercial kits in each group. Data were shown as mean ± SEM. Statistical significance was determined using two-tailed unpaired Student’s *t*-test (**A**–**C**) and one-way ANOVA followed by post hoc Bonferroni test (**D**–**G**). n = 12 slices from 6 rats, two slices per rat (**A**). n = 3 rats per group (**B**). n = 3 of independent cell culture preparations (**C**, **E**). n = 6 of independent cell culture preparations (**G**). n = 12 slices from 6 of independent cell culture preparations, two slices per independent cell culture preparation (**D**, **F**). *p < 0.05 vs. SIH group. ^#^p < 0.05, ^##^p < 0.01  vs. TNF-α group

Sirt3 deficiency has been found to be associated with mitochondrial dysfunction in many diseases [44]. Immunofluorescence and Western blot experiments were performed to assess the Sirt3 expression in the RVLM of SIH and control rats. The results revealed that the expression of Sirt3 was significantly reduced in tyrosine hydroxylase (TH)-positive neurons in the RVLM of the SIH group compared with the control group (Fig. 5A). The protein level of Sirt3 was remarkably reduced in the RVLM of SIH rats (Fig. 5B). Compared with the control group, the p‐AMPK expression in the RVLM of the SIH group had significantly decreased, indicating that the AMPK pathway was inactivated (Fig. 5B). Importantly, the expressions of p‐AMPK and Sirt3 were remarkably downregulated in N2a cells after TNF‐α treatment (Fig. 5C). N2a cell viability was repressed by TNF-α but sustained by AMPK activator A769662 treatments (30 and 60 μM) as assessed via CCK-8 assay (Additional file 1: Fig. S1). AMPK pathway activation using A769662 rescued p-AMPK and Sirt3 expression in TNF‐α–treated N2a cells (Additional file 2: Fig. S2). Next, we explored the potential effect of AMPK–Sirt3 pathway activation in attenuating TNF-α–mediated mitochondrial dysfunction. Mitochondrial membrane potential (MMP) was inhibited by TNF‐α exposure in N2a cells, whereas A769662 reversed MMP depolarization (Fig. 5D). As shown in Fig. 5E, AMPK activation also eliminated the inhibitory effect of TNF‐α on mitochondrial respiratory metabolism as evidenced by the enhanced protein levels of mitochondrial respiratory chain complexes (complexes II-SDHB, III-UQCRC2, and V-ATP5A). Compared to the control group, ROS production was upregulated whereas SOD and CAT activities were downregulated upon TNF-α treatment, and these effects could be reversed by A769662 administration (Fig. 5F, G). The above findings illustrated that Sirt3 might be inhibited by microglia-derived TNF-α in an AMPK‐dependent manner and contributed to neuronal mitochondrial dysfunction.

### Microglia-derived TNF-α blockade attenuated RVLM neuronal mitochondrial injury in SIH rats

**Fig. 6:**
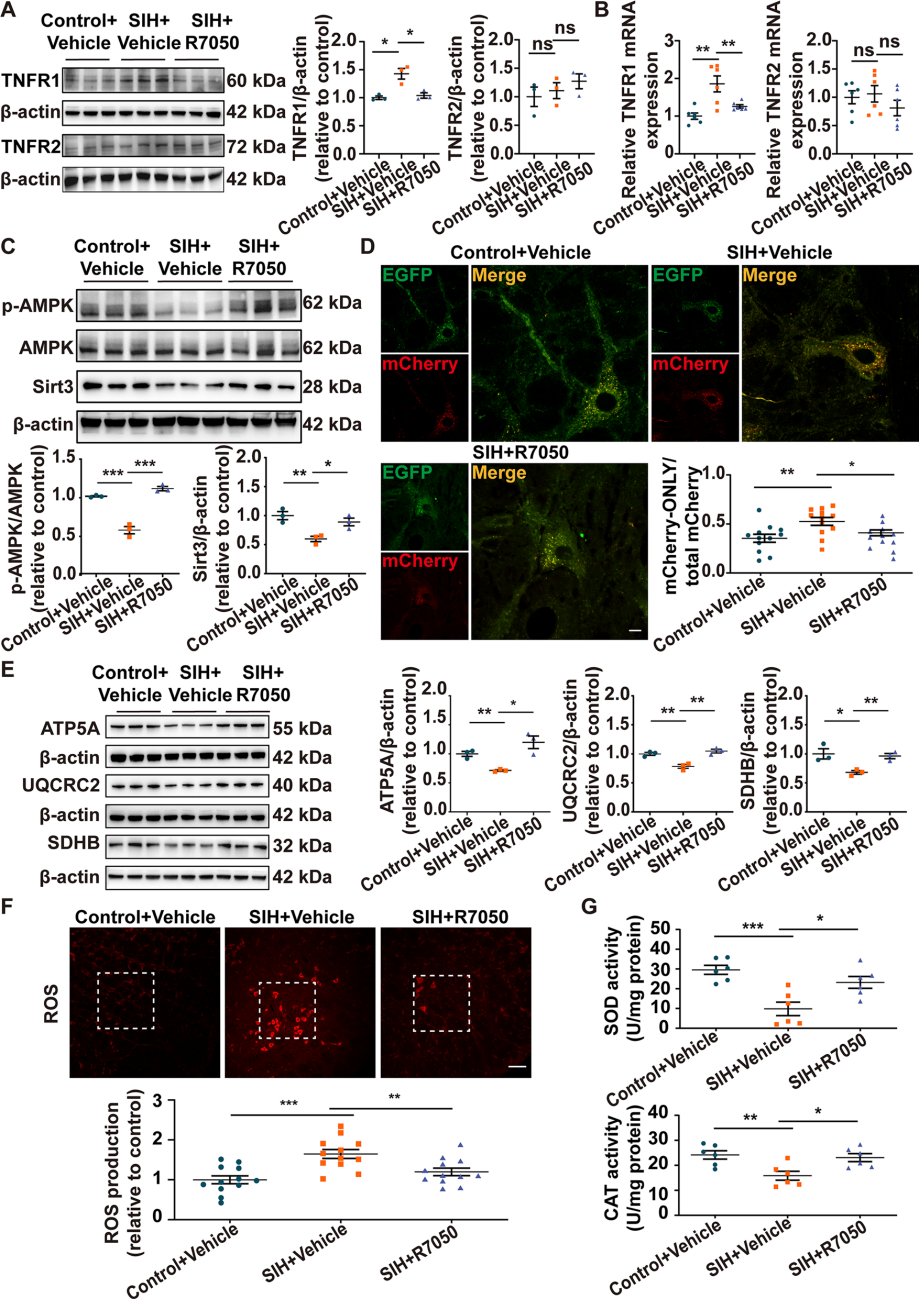
Blockage of TNF-α rescued mitochondrial injury in the RVLM neurons of SIH rats. The expression of **A** TNFR1 and **B** TNFR2 was determined by Western blot and RT-qPCR assays in the RVLM of control + vehicle, SIH + vehicle, and SIH + R7050 rats. **C** Representative immunoblot bands and quantitative analysis showed the expression of p-AMPK and Sirt3 in the RVLM between the three groups. **D** Representative images of MitoEGFPmCherry labeling in the RVLM of different groups, and quantitative analysis of the degree of mitochondrial injury was evaluated as the ratio value of mCherry-ONLY/total mCherry puncta. Scale bar = 10 μm. **E** Representative immunoblot bands and quantitative analysis of mitochondrial respiratory chain complexes (complexes II-SDHB, III-UQCRC2, and V-ATP5A) in the RVLM in control + vehicle, SIH + vehicle, and SIH + R7050 groups. **F** Representative fluorescence images of reactive oxygen species (ROS) detection and statistical data of ROS production in the RVLM between the three groups. Scale bar = 100 μm. **G** The levels of superoxide dismutase (SOD) and catalase (CAT) were quantified by using commercial kits in each group. Data were shown as mean ± SEM. Statistical significance was determined using one-way ANOVA followed by post hoc Bonferroni test (**A**–**G**). n = 3 rats per group (**A**, **C**, **E**). n = 6 rats per group (**B**, **G**). n = 12 slices from 6 rats, two slices per rat (**D**,** F**). *p < 0.05, **p < 0.01, ***p < 0.001 vs. SIH + Vehicle group. ns means nonsignificant vs. SIH + Vehicle group

The biological functions of TNF-α are typically initiated by its engagement with TNF-α receptors (TNFR1 and TNFR2) [45]. The RVLM of rats was microinjected bilaterally with the TNF-α receptor antagonist R7050 on 10 and 15 days during chronic stress to block the effect of microglia-derived TNF-α (Fig. 1). Western blot and RT-qPCR results showed that R7050 administration remarkably inhibited the increase in TNFR1 protein and mRNA levels in the RVLM of SIH rats. By contrast, R7050 had no effect on TNFR2 expression (Fig. 6A, B). TNF-α suppression reversed the downregulation of p-AMPK and Sirt3 in the SIH group (Fig. 6C). Subsequently, we investigated whether the inhibition of TNF-α could rescue injured mitochondria within RVLM neurons of SIH rats. The MitoEGFPmCherry reporter was used to detect the degree of mitochondrial degradation in the RVLM. Compared with the SIH + Saline group, the group treated with R7050 had significantly decreased number of mCherry-ONLY signal (Fig. 6D). Compared with the control group, the protein levels of the mitochondrial respiratory chain complexes (complexes II-SDHB, III-UQCRC2, and V-ATP5A) were markedly decreased in the SIH group, and R7050 administration reversed these effects (Fig. 6E). Figure 6F, G revealed that R7050 treatment considerably decreased ROS production and improved the activities of mitochondrial antioxidant factors, as evidenced by increased SOD and CAT activities. These results suggested that TNF-α inhibition reversed mitochondrial injury in the RVLM neurons of SIH rats.

### Inhibition of TNF-α in the RVLM decreased neuronal excitation, sympathetic outflow, BP, and HR in SIH rats

**Fig. 7:**
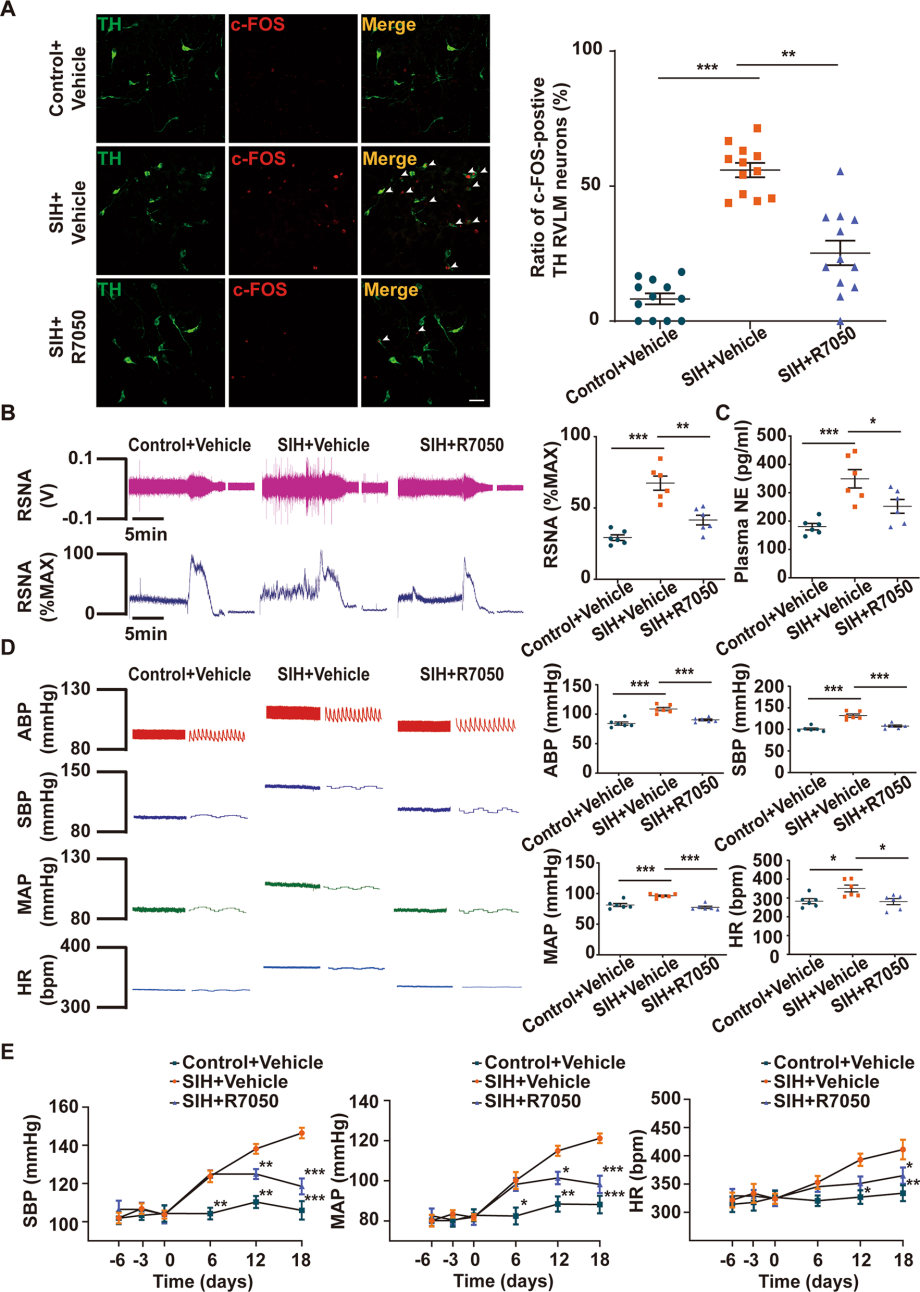
TNF-α inhibition in the RVLM reduced neuronal overactivation, sympathetic outflow, blood pressure (BP), and heart rate (HR) in SIH rats. **A** Representative immunofluorescence images and quantitative analysis showed colocalization of c-FOS and tyrosine hydroxylase (TH)-positive neurons in the RVLM of control + vehicle, SIH + vehicle, and SIH + R7050 groups. Scale bar = 50 μm. **B** Renal sympathetic nerve activity (RSNA) recording and **C** plasma norepinephrine (NE) ELISA assay were performed in the three groups. **D** The arterial blood pressure (ABP), systolic blood pressure (SBP), mean arterial pressure (MAP), and HR were tested in anesthetized rats. **E** The levels of SBP, MAP and HR were measured by telemetry in conscious rats. Data were shown as mean ± SEM. Statistical significance was determined using one-way ANOVA followed by post hoc Bonferroni test (**A**–**E**). n = 12 slices from 6 rats, two slices per rat (**A**). n = 6 rats per group (**B**–**E**). *p < 0.05, **p < 0.01, ***p < 0.001 vs. SIH + Vehicle group

Double immunofluorescent staining of neuronal activation marker c-FOS with TH-positive neurons in the RVLM was performed to study neuronal excitability. The result showed that TNF-α suppression could significantly decrease the proportion of c-FOS in TH-positive neurons in the RVLM of SIH rats (Fig. 7A). Subsequently, we investigated the protective effect of TNF-α inhibition in the RVLM on alleviating sympathetic hyperactivity in SIH rats. Results showed that R7050 application considerably downregulated RSNA in SIH rats (Fig. 7B). The level of plasma NE was also decreased in the SIH + R7050 group (Fig. 7C). Furthermore, the HR, ABP, SBP, and MAP levels of the SIH rats were markedly reduced by R7050 administration as assessed by femoral artery cannulation and telemetry (Fig. 7D, E). Therefore, these data confirmed that the blockage of TNF-α in the RVLM reduced neuronal overactivation, sympathetic hyperactivity, BP, and HR in SIH rats.

## Discussion

The main purpose of this study was to investigate the mechanism of microglial neuroinflammation affecting mitochondrial homeostasis in the rostral ventrolateral medulla (RVLM) neurons involved in the pathology of SIH. Our major findings were as follows: (1) Increased BP, HR, sympathetic outflow, and RVLM neuronal overactivity were exhibited in SIH rats; (2) Microglia were activated and particularly polarized to a M1 proinflammatory phenotype, accompanied by increased level of TNF-α in the RVLM of SIH rat; (3) The RVLM neuronal mitochondria were evidently injured in SIH rats; (4) Microglia-derived TNF-α resulted in neuronal mitochondrial dysfunction via the AMPK–Sirt3 signaling pathway and; (5) Microglia-derived TNF-α inhibition in the RVLM could rescue the injury of neuronal mitochondria and inhibit neuronal overactivation, sympathetic hyperexcitability, BP, and HR in SIH rats. Collectively, the present study revealed that microglia-derived TNF-α impaired RVLM neuronal mitochondrial function via the dysregulation of the AMPK–Sirt3 signaling pathway and that microglia-derived TNF-α might be a novel target for therapeutic intervention in SIH.

Neuroinflammation is implicated in the pathophysiology of many neurological diseases, including traumatic brain injury, mental illness, and neurodegenerative diseases [46]. Microglia, commonly known as brain-resident immune cells, are primary regulators of neuroinflammation [47]. Chronically activated microglia can produce proinflammatory cytokines to promote neuroinflammatory responses and exert detrimental effects on neuronal health [48]. Microglia-derived proinflammatory cytokine TNF-α has been confirmed to contribute to neuronal cell cycle events in the pathogenesis of Alzheimer’s disease [49]. A previous study has demonstrated that TNF-α released by activated microglia enhanced glutamatergic transmission of substantia gelatinosa neurons involved in the development of neuropathic pain [50]. Yamamoto et al*.* proposed TNF-α secretion from activated microglia triggered the increase of cerebellar neuronal excitability and modulated psychomotor behaviors [51]. The microglia-mediated neuroinflammation has been linked to SIH [17]. In the present study, morphological alterations in microglia with reduced ramifications and complexity have been observed in the RVLM of SIH rats compared with the control rats, and this finding is indicative of microglial activation. Western blot analysis demonstrated that activated microglia in the RVLM of SIH rats were highly polarized into M1 phenotype. The immunofluorescence co-localization analysis showed that TNF-α was derived from the microglia. Moreover, the protein and mRNA levels of TNF-α were remarkably increased in the RVLM of SIH rats. These results suggested that microglia-derived TNF-α has a remarkable correlation with the etiology of SIH.

Structurally damaged mitochondria are dysfunctional and unable to maintain the normal physiological activities of neurons [52]. In this study, the RVLM neuronal mitochondria were swollen, and the mitochondrial integrity was disrupted in SIH rats. According to the MitoEGFPmCherry reporter, a significantly higher proportion of mitochondria was degraded in the RVLM neurons of SIH rats than that of control rats. Moreover, SIH rats exhibited markedly impaired mitochondrial respiratory metabolism and enhanced mitochondrial oxidative stress level. Thus, exploring the causes of mitochondrial injury in the RVLM neurons is crucial to understanding the pathogenesis of SIH. Recent studies have shown that excessive inflammatory response is associated with neuronal mitochondrial impairment in the progression of illnesses [53]. The elevated microglia-derived TNF-α levels in the RVLM might be a key factor in neuronal mitochondrial defects of SIH rats. The present study reported the treatment of TNF-α in vitro significantly reduced mitochondrial membrane potential (MMP) and respiratory metabolism and caused mitochondrial oxidative stress damage in N2a cells. By contrast, the in vivo microglia-derived TNF-α inhibition had protective effects on mitochondrial function in SIH rats, manifested by increased expression levels of mitochondrial respiratory chain complexes, enhanced superoxide dismutase (SOD) and catalase (CAT) activities, and decreased ROS levels and mCherry-ONLY signal. These findings suggested that microglia-derived TNF-α disturbed RVLM neuronal mitochondrial homeostasis and participated in the occurrence and development of SIH.

Sirt3 has been substantiated to modulate almost all aspects of mitochondrial metabolism and function, protecting mitochondria from various injuries [54, 55]. Current results revealed a lower level of Sirt3 expression in the RVLM of SIH rats than in healthy controls, showing that microglia-derived TNF-α–induced the reduction of neuronal mitochondrial function is associated with Sirt3 dysregulation. The aberrant AMPK–Sirt3 signaling is responsible for mitochondrial dysfunction involved in the various disease processes. A recent study has reported that AMPK may regulate Sirt3 and participate in the inhibitory effect of celastrol on inflammation [56]. Wang et al*.* reported that honokiol attenuated fluoride-induced mitochondrial injury and neuronal/synaptic impairment, which depended on promoting the expression of Sirt3 via an AMPK signaling pathway [57]. Yu et al*.* revealed that the protective actions of naringenin on mitochondrial dysfunction and cardiac damage following ischemia–reperfusion injury were abolished by the blockage of the AMPK–Sirt3 signaling pathway [58]. Activation of the AMPK–Sirt3 pathway reduces the mitochondrial ROS accumulation in vascular endothelium by augmenting SOD activity [59]. The present study demonstrated that the AMPK pathway was inactivated in the RVLM of the SIH group, as evidenced by decreased AMPK phosphorylation. Meanwhile, we found that TNF-α stimulation resulted in a marked reduction of AMPK phosphorylation and Sirt3 in N2a cells. Interestingly, AMPK activator A769662 promoted AMPK phosphorylation and thus triggered the upregulation of Sirt3 and reversed mitochondrial dysfunction induced by TNF-α, suggesting that the protective effect of AMPK–Sirt3 signaling pathway activation against TNF-α–mediated mitochondrial injury. TNF-α exerts multiple functions in the nervous system through binding to its two main receptors: TNFR1 and TNFR2 [60]. The blockage of the integration of TNF-α with its receptor, TNFR1, eliminates TNF-α–induced detrimental effects [61]. Previous investigators have shown that TNF-α receptor inhibitor R7050 possesses neuroprotective and anti-inflammatory effects [62]. Ding et al*.* reported that R7050 reversed TNF-α-induced increase in NADPH oxidase activity in rats with obesity-related hypertension [37]. In the present study, administration of R7050 into the RVLM significantly decreased the level of TNFR1 in SIH rats. Furthermore, the biological function of TNF-α suppression increased the expression of AMPK phosphorylation and Sirt3 and consequently alleviated neuronal mitochondrial injury in the RVLM of SIH rats. Taken together, microglia-derived TNF-α in the RVLM caused neuronal mitochondrial dysfunction in SIH, possibly through blocking the AMPK–Sirt3 pathway.

Chronic stress, which can be perceived by the brain, contributes to the development of SIH [63]. Stress promotes RVLM neuronal hyperactivity and further excites the sympathetic nerve; this seems to be the major factor in SIH progression. The SIH rat model is established by electric foot-shock stressors with noise interventions, which is proposed as a reliable model for exploring the pathogenesis of SIH [6, 16, 17, 64, 65]. This study showed that microglia-derived TNF-α has a crucial role in the progression of SIH, indicating that TNF-α blockade may be an effective intervention in the treatment of SIH. The present results showed that TNF-α inhibition by microinjection of R7050 attenuated RVLM neuronal overactivation and decreased sympathetic hyperexcitability, BP, and HR in SIH rats. The suppression of microglia-derived TNF-α in the RVLM exerts anti-hypertensive effects, but its roles in other cardiovascular control regions, such as the hypothalamic paraventricular nucleus (PVN) and the nucleus tractus solitarius (NTS), have not been investigated. Therefore, the role and importance of microglia-derived TNF-α in these central control regions will be further explored in the coming years.

## Conclusion

**Fig. 8:**
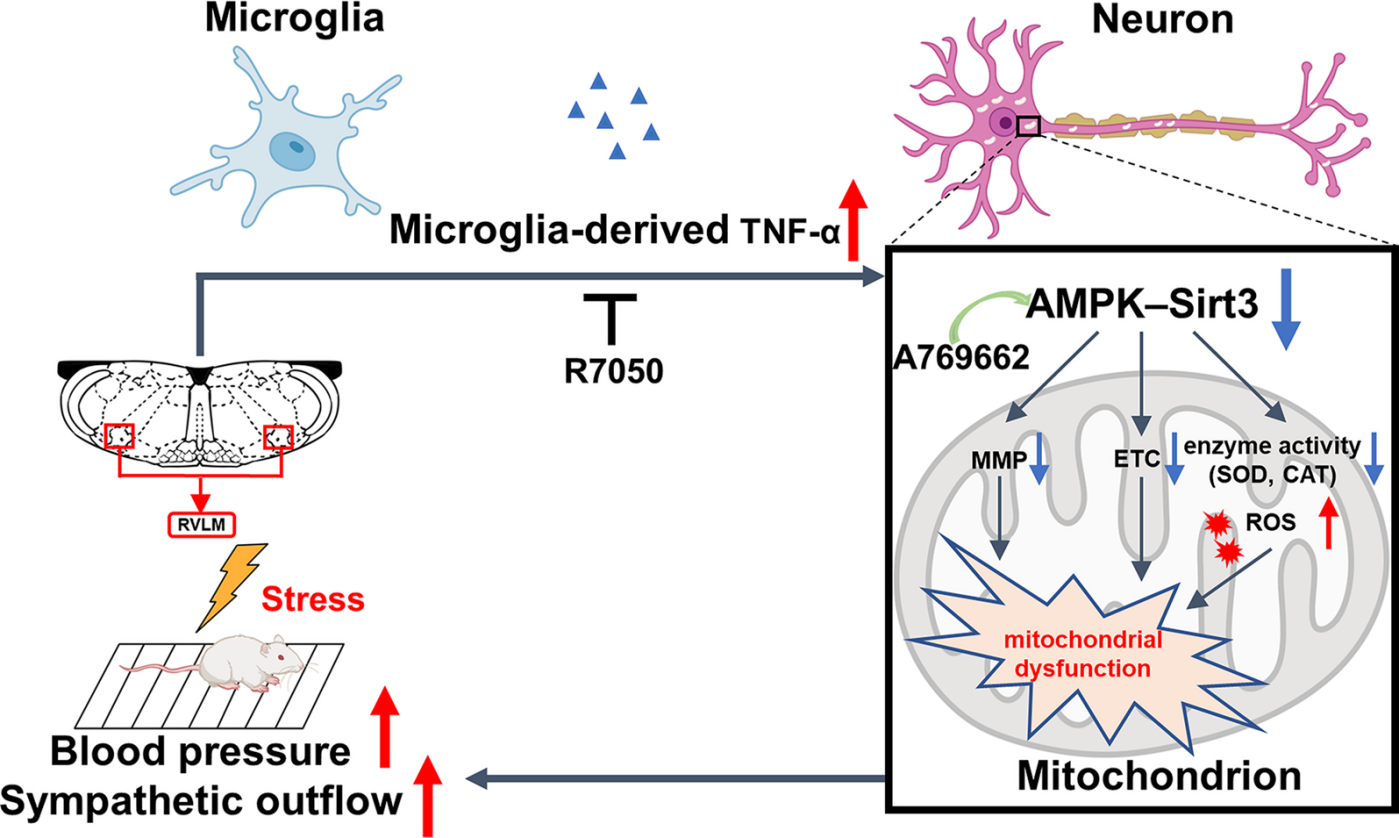
Schematic diagram illustrating the mechanisms of the pressor effect of microglia-derived TNF-α via impairing neuronal mitochondrial function in the RVLM. A769662: AMPK activator; CAT: catalase; ETC: electron transport chain; MMP: mitochondrial membrane potential; R7050: TNF-α receptor antagonist; ROS: reactive oxygen species; SOD: superoxide dismutase; TNF-α: tumor necrosis factor-α

This study indicated that elevated TNF-α, which was derived from activated microglia, could cause neuronal hyperexcitation via disturbing mitochondrial homeostasis in the RVLM of SIH rats (Fig. 8). The results further confirmed that microglia-derived TNF-α induced mitochondrial dysfunction by blocking the AMPK–Sirt3 pathway. TNF-α suppression provides a protective role against neuronal mitochondrial injury and alleviates neuronal excitation, sympathetic outflow, BP, and HR. Accordingly, microglia-derived TNF-α may be an avenue for therapeutic target for the intervention of SIH.

## Supplementary Information

**Additional file 1: Figure S1.** Cell viability was determined using the cell counting kit-8  assay after treatment with TNF-α or TNF-α plus AMPK activator A769662 in N2a cells. Data were shown as mean ± SEM. Statistical significance was determined using one-way ANOVA followed by post hoc Bonferroni test. n = 6 of independent cell culture preparations. #p < 0.05, ##p < 0.01 *vs*. TNF-α group.

**Additional file 2: Figure S2.** The AMPK pathway activation rescued p‐AMPK and Sirt3 levels in TNF‐α-treated N2a cells. Representative immunoblot bands and quantitative analysis of Sirt3 and p-AMPK expression in control, TNF-α, and TNF-α plus AMPK activator A769662 groups. Data were shown as mean ± SEM. Statistical significance was determined using one-way ANOVA followed by post hoc Bonferroni test. n = 3 of independent cell culture preparations. #p < 0.05, ##p < 0.01, ###p < 0.001 *vs*. TNF-α group.

## Data Availability

All data generated in this study are included in this manuscript.

## Abbreviations

ABP: Arterial blood pressure
BP: Blood pressure
Cyt-cyto C: Cytoplasmic cytochrome C
EEG: Electroencephalogram
ELISA: Enzyme-linked immunosorbent assay
HR: Heart rate
MAP: Mean arterial pressure
MMP: Mitochondrial membrane potential
NE: Norepinephrine
NTS: Nucleus tractus solitarius
PSD: Power spectral density
PVN: Hypothalamic paraventricular nucleus
ROS: Reactive oxygen species
RSNA: Renal sympathetic nerve activity
RT-qPCR: Reverse transcription quantitative polymerase chain reaction
RVLM: Rostral ventrolateral medulla
SBP: Systolic blood pressure
SEM: Standard error of the mean
SIH: Stress-induced hypertension
Sirt3: Sirtuin-3
TEM: Transmission electron microscopy
TNF‐α: Tumor necrosis factor‐α
TH: Tyrosine hydroxylase
