# Supplementary figures and images for: Microglia-derived TNF-α contributes to RVLM neuronal mitochondrial dysfunction via blocking the AMPK–Sirt3 pathway in stress-induced hypertension
Source: J Neuroinflammation. 2023 Jun 1;20:137. doi: 10.1186/s12974-023-02818-6 (PMC10236846; doi:10.1186/s12974-023-02818-6)

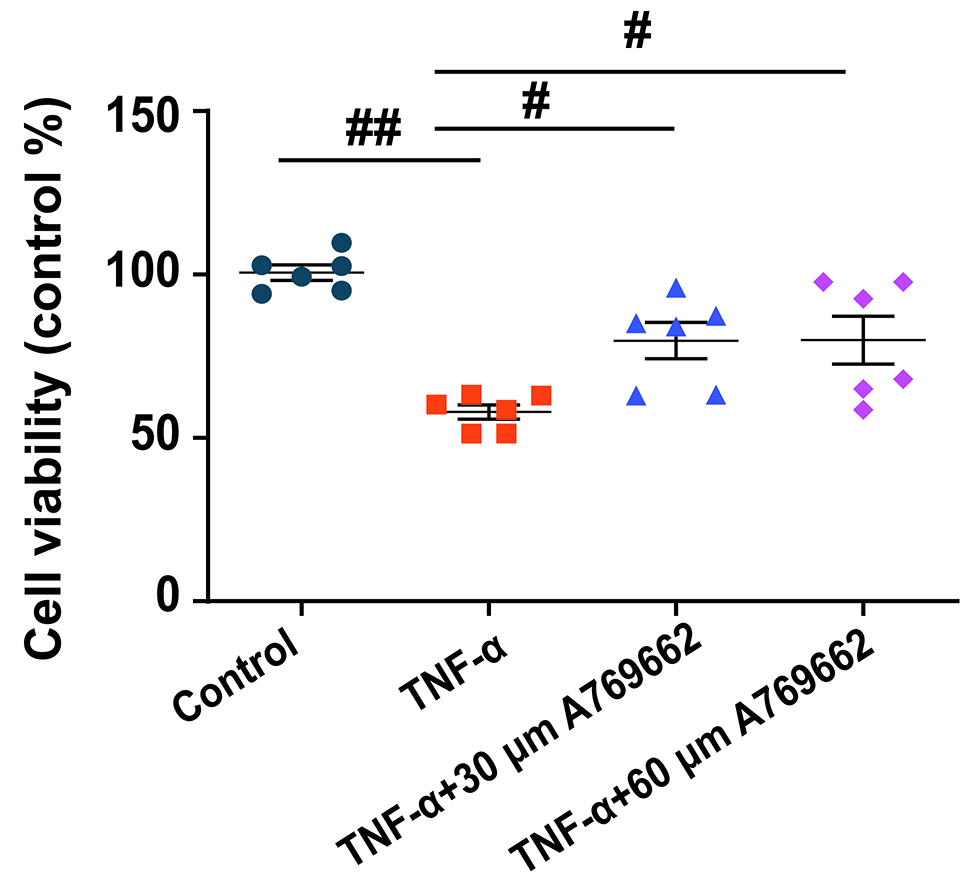

Supplement: Supplementary file 1 — Additional file 1: Figure S1. Cell viability was determined using the cell counting kit-8 assay after treatment with TNF-α or TNF-α plus AMPK activator A769662 in N2a cells. Data were shown as mean ± SEM. Statistical significance was determined using one-way ANOVA followed by post hoc Bonferroni test. n = 6 of independent cell culture preparations. #p < 0.05, ##p < 0.01 vs. TNF-α group. [file 12974_2023_2818_MOESM1_ESM.tif]

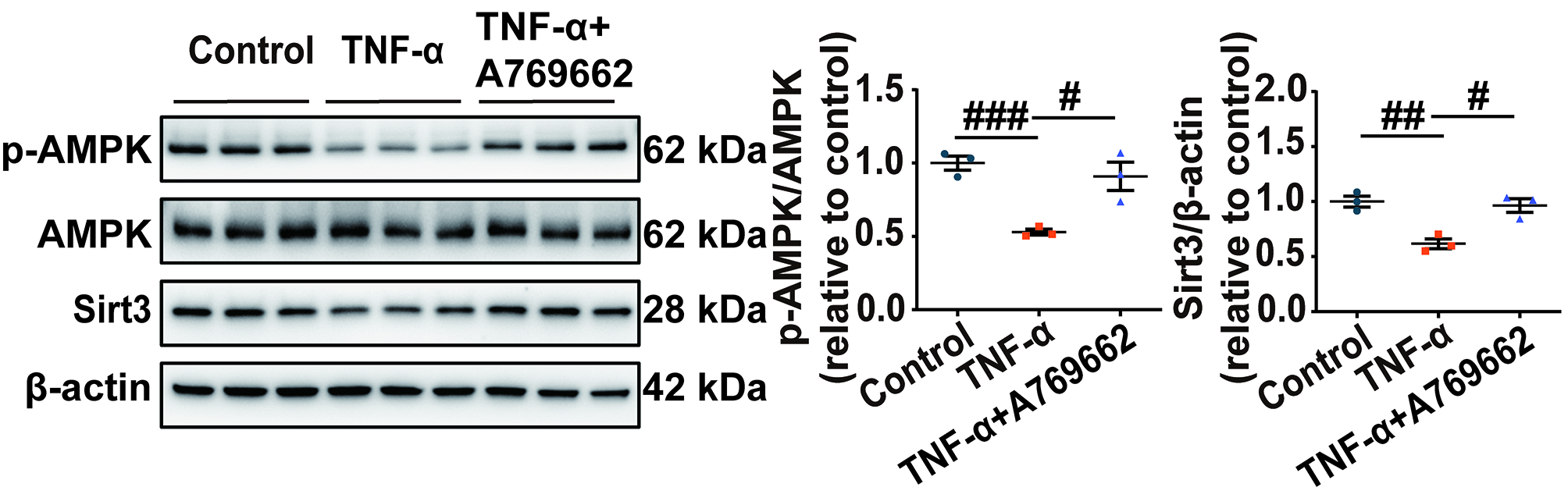

Supplement: Supplementary file 2 — Additional file 2: Figure S2. The AMPK pathway activation rescued p‐AMPK and Sirt3 levels in TNF‐α-treated N2a cells. Representative immunoblot bands and quantitative analysis of Sirt3 and p-AMPK expression in control, TNF-α, and TNF-α plus AMPK activator A769662 groups. Data were shown as mean ± SEM. Statistical significance was determined using one-way ANOVA followed by post hoc Bonferroni test. n = 3 of independent cell culture preparations. #p < 0.05, ##p < 0.01, ###p < 0.001 vs. TNF-α group. [file 12974_2023_2818_MOESM2_ESM.tif]
